# In Search of Biomarkers for Idiopathic Scoliosis: *Leptin* and *BMP4* Functional Polymorphisms

**DOI:** 10.1155/2015/425310

**Published:** 2015-08-02

**Authors:** Svetla Nikolova, Vasil Yablanski, Evgeni Vlaev, Gergana Getova, Ventseslav Atanasov, Luben Stokov, Alexey Slavkov Savov, Ivo Marinov Kremensky

**Affiliations:** ^1^National Genetic Laboratory, Department of Obstetrics and Gynecology, Faculty of Medicine, Medical University, Sofia, 2 Zdrave Street, 14 Floor, 1431 Sofia, Bulgaria; ^2^Tokuda Hospital Sofia, Orthopedic and Traumatology Clinic, 51B Nikola Vaptsarov Boulevard, 1407 Sofia, Bulgaria; ^3^University Orthopedic Hospital “Professor Boycho Boychev”, Medical University, Sofia, 56 Nikola Petkov Boulevard, 1614 Sofia, Bulgaria; ^4^Molecular Medicine Center, Medical University, Sofia, 2 Zdrave Street, 14 floor, 1431 Sofia, Bulgaria

## Abstract

Idiopathic scoliosis (IS) is the most common spinal disorder in children and adolescents. The current consensus on IS maintains that it has a multifactorial etiology with genetic predisposition factors. In the present study the association of two functional polymorphisms of *leptin* (rs7799039) and *BMP4* (rs4898820) with susceptibility to IS and curve severity was investigated in a Bulgarian population sample. The molecular detection of the genotypes was performed by amplification followed by restriction technology. The statistical analysis was performed by Pearson's chi-squared test. This case-control study revealed no statistically significant association between the functional polymorphisms of *leptin* and *BMP4* and susceptibility to IS or curve progression (*p* > 0.05). On the basis of these results the examined polymorphic variants of *leptin* and *BMP4* could not be considered as genetic variants with predisposition effect or as risk factors for the progression of the curve. In addition, these results do not exclude a synergistic effect of the promoter polymorphisms of *leptin* and *BMP4* in the etiology and pathogenesis of IS. The identification of molecular markers for IS could be useful for early detection and prognosis of the risk for a rapid progression of the curve. That would permit early stage treatment of the patient with the least invasive procedures.

## 1. Introduction

Idiopathic scoliosis (IS) is the most common spinal disorder in children and adolescents [1]. It is characterized by lateral deviation of the spine of more than 10 degrees combined with a rotation of the vertebrae [1, 2]. The current consensus on IS maintains that it has a multifactorial etiology with genetic predisposition factors.

Leptin and its signaling pathway may be a candidate for the etiology of IS. Leptin, together with the soluble leptin receptor (sOB-R), was shown to play an important role in the regulation of bone and energy metabolism in children. Leptin affects bone metabolism via central and peripheral ways. It modulates cortical bone formation by regulating the expression of several neuropeptides in hypothalamus and inducing sympathetic activation [3–5]. It also directs the bone marrow stromal cells to the osteogenic instead of the adipogenic pathway [6, 7]. Thus an abnormal leptin level or the deficiency of signal pathway may result in a disorder in skeletal growth.

Qiu et al. [8] showed that girls with adolescent idiopathic scoliosis (AIS) have lower leptin levels than the controls, and the decreased leptin levels are associated with lower body mass and bone mass. Liu et al. [9] demonstrated the presence of abnormal leptin bioavailability in girls with AIS. According to Tam et al. [10] the osteoblasts isolated from AIS girls have very low response to leptin compared with controls.

The* leptin* gene is considered as a candidate gene for IS. The 2548G/A leptin promoter polymorphism is a common single nucleotide substitution which could possibly be associated with serum leptin concentration or body mass index [11–14]. The functional G-2548A polymorphism (rs7799039) of* leptin* was associated with numerous diseases [15–19]. The association study conducted by Mórocz et al. [20] was the first one linking the functional polymorphism G-2548A of* leptin* to susceptibility to IS. They found no single association but investigated the interaction between the* leptin* (rs7799039) and the* IL-6* (rs1800795) genetic polymorphisms and concluded that the OR of the AA-(*Lep*)-CC-(*IL6*) double homozygote genotype combination was found to be significantly elevated in patients with AIS (*p* = 0.027; OR = 4.67; CI: 1.24–17.60).

Liang et al. [2] found no association between six other single nucleotide polymorphisms (rs3828942 in intron, rs75506045 in a coding region, rs10954174, rs41457646 and rs11761556 in the untranslated 3′-region, and one unlabelled polymorphism in a coding region) of the* leptin* and genetic susceptibility/severity of AIS. The* leptin* gene expression was normal in AIS induced adipocytes and osteoblasts after an adjustment of the differentiation rate. The decreased expression of leptin receptor in AIS girls may lead to hyposensitivity to leptin according to the authors. The abnormal response to leptin may have a role in the pathogenesis of AIS in Chinese population [2].

The bone morphogenetic protein 4 (BMP4) is a vital regulatory molecule that functions throughout the development in mesoderm induction, tooth development, limb formation, bone induction, and fracture repair (OMIM, 112262). Mórocz et al. [20] found no single association between AIS and* BMP4* (rs4898820, located in 5′ upstream of the gene) in the study of Hungarian population but investigated the interaction between the* BMP4* (rs7799039) and the* MMP3* (rs3025058) functional genetic polymorphisms and concluded the ORs of the GG-5A/5A, GG-5A/6A, TT-6A/6A, GT-5A/5A, GT-5A/6A, and GT-6A/6A showed significantly elevated ORs (OR from 5.89 to 7.8).

In the present study the association of two functional polymorphisms of* leptin* (rs7799039) and* BMP4* (rs4898820) with susceptibility to IS and curve severity was investigated in a Bulgarian population sample.

## 2. Materials and Methods

### 2.1. Subject

Patients with IS (*n* = 90) were recruited with the help of orthopaedic surgeons from Tokuda Hospital Sofia and University Orthopedic Hospital “Professor Boycho Boychev.” The IS diagnosis was confirmed clinically and radiologically. The curves were measured by the Cobb method. The mean value of Cobb angles was 54.6 ± 23.2. The mean age at the beginning of the disease was 11.2 ± 3.1 years.

The patients were grouped by 5 indices: (1) age of the onset of the disease; (2) familial history; (3) Cobb's angle; (4) curve pattern; and (5) gender.

IS was observed in three age groups: infantile, from one to three years of age (*n* = 4), juvenile, from greater than three years of age up to nine years of age (*n* = 18), and adolescent from ten to sixteen years of age (*n* = 68).

Patients with positive familial history (*n* = 23) and sporadic cases (*n* = 67) were included.

The Cobb angle under consideration was either the final Cobb angle, defined as the curve angle in skeletally mature patients, or the Cobb angle, measured in the last follow-up of those patients who were skeletally immature. Patients were assigned to two treatment groups: one surgical (*n* = 70) with a Cobb angle of 40 degrees or higher and one nonsurgical (*n* = 20).

One curve is usually greater than the other(s) and is referred to as the major curve. Concerning the major curve there are four common types of curve patterns associated with scoliosis: thoracic, lumbar, thoracolumbar, and double major curve pattern. The curve(s) of lesser degree is/are considered compensatory (secondary or minor) curve(s). The most common type of such balanced scoliosis is the S-shaped scoliosis. Two curves may be of equal size and thus are considered a double major curve pattern. Patients with thoracic (*n* = 56), lumbar (*n* = 8) and thoracolumbar (*n* = 26) curve pattern and S-shaped scoliosis (*n* = 28) were included.

In this study, male (*n* = 16) and female (*n* = 74) patients were included.

The control group (*n* = 180) including healthy subjects without clinical signs of IS was recruited from a pool of unrelated gender-matched volunteers from other Units and Clinics of Tokuda Hospital Sofia, National Genetic Laboratory, hospital staff members and students. The controls were selected among adult patients with skeletal maturity with negative family history of IS. Radiological examination was not performed in the control group.

Peripheral blood samples were obtained from all patients and control subjects. The study protocol was approved by the Ethics Committee of the Medical University, Sofia. All the involved patients, or their parents in case of children, and all volunteers provided written informed consent.

### 2.2. Genotyping

Genomic DNA was extracted from the peripheral blood leucocytes using magnetic bead technology (chemagic DNA Blood Kit special, Chemagen, Germany) on automated high throughput nucleic acid isolation platform (chemagic Magnetic Separation Module I, Chemagen, Germany).

The polymorphic regions of* leptin* and* BMP4* genes were amplified by polymerase chain reaction (PCR) with primer sets previously designed by Mórocz et al. [20]. The PCR was carried out in a reaction mix of 20 *μ*L containing 100 ng DNA and 10x Prime Taq buffer (Genet Bio, Korea), 10 mM dNTPs mixture (Genet Bio, Korea), 20 pmol forward and reverse primers (AlphaDNA, Canada), and 0.1 U Prime Taq DNA polymerase (Genet Bio, Korea). PCR amplifications were performed in an AB 2720 Thermocycler (Life Technologies, USA) with an initial denaturation at 94°C for five minutes and a final extension of seven minutes at 72°C. The following thermal cycle was repeated 30 times: denaturation at 94°C for 30 seconds, annealing at 55°C for* leptin* and 59°C for* BMP4* for 30 seconds, and extension at 72°C for 30 seconds.

The PCR products were cleaved with the restriction enzymes:* Hha*I (NEB, USA) for* leptin* and* Mlu*CI (NEB, USA) for* BMP4*, according to the manufacturer's instructions, and the obtained fragments were separated on agarose 3% gel in VG-SYS Horizontal Electrophoresis System (Biochrom, UK).

### 2.3. Statistical Analysis

The statistical analysis was performed by *χ* ^2^ test to make genotype and allele comparisons between cases and controls as well as test for Hardy-Weinberg equilibrium. A value of *p* < 0.05 was considered to be statistically significant for comparison between data sets. Statistical analysis was conducted using SPSS version 19.0 (IBM, NY).

## 3. Results

This study examined the association between IS and two functional polymorphisms: the* leptin* promoter polymorphism at -2548 position (rs7799039 G/A) and the* BMP4* promoter polymorphism at -3445 position (rs4898820 G/T). Genotypes were in Hardy-Weinberg equilibrium.

The genotype and allele frequencies of the case and the control group are summarised in Table 1.

The overall frequencies of the AA and the GG genotype of the* leptin* -2548 G/A polymorphism in the patients with IS were comparable with the controls (AA, 27.8 versus 30.6% and GG, 25.6 versus 20.6%, *p* = 0.64). In conclusion, a specific genotype was not associated with a higher risk of scoliosis (AA versus GG, OR: 1.37; 95% CI: 0.68–2.76) and the presence of a given allele (A versus G, OR: 1.17, 95% CI: 0.82–1.67) could not be considered as a susceptibility factor to IS.

The frequencies of the GG and the TT genotype of the* BMP4* -3445 G/T polymorphism in the IS patients were also comparable with the control subjects (TT, 30.0 versus 25.6% and GG, 18.9 versus 19.4%, *p* = 0.74). In conclusion, a specific genotype was not associated with a higher risk of scoliosis (TT versus GG, OR: 1.21; 95% CI: 0.57–2.56) and the presence of a given allele (T versus G, OR: 1.11, 95% CI: 0.77–1.58) could not be considered as a susceptibility factor to IS.

We separated the cases in subgroups according to age, gender, Cobb angle, curve pattern, and familial history of IS and then investigated the associations in the different subgroups. Odds ratios of genotypes and alleles in the subgroups are summarised in Table 2.

In the subgroup of the surgical cases where Cobb angle was 40 degrees or higher the genotype and allele frequencies of the* leptin* promoter polymorphism and the* BMP4* promoter polymorphism were comparable between cases and controls (AA versus GG, *p* = 0.63; OR: 1.2; 95% CI: 0.56–2.58 and TT versus GG, *p* = 1; OR: 1.01; 95% CI: 0.46–2.26).

In the subgroup of the familial cases, elevated ORs were observed (AA versus GG, OR: 2.08; 95% CI: 0.61–7.06 and TT versus GG, OR: 3.42; 95% CI: 0.7–16.86) but the 95% CI included 1.0 (Table 2).

In the subgroup of AIS, the genotype and allele frequencies of the* leptin* and the* BMP4* promoter polymorphisms were comparable between cases and controls (*p* > 0.05).

No genotype or allele of the* leptin* and the* BMP4* promoter polymorphisms was found to be correlated with age, gender, or curve pattern (Table 2).

## 4. Discussion

Predisposition for IS, like other examples of complex traits, does not have a specific assigned risk of heritability, but inheritance is based on multiple factors, potentially both genetic and environmental [21].

In our study, the frequencies of the genotypes of the* leptin* -2548 G/A polymorphism (rs7799039) and the* BMP4* -3445 G/T polymorphism (rs4898820) showed no statistically significant differences between cases and controls (*p* > 0.05). On the basis of these results the genetic variants of* leptin* and* BMP4* alone could not be considered as predisposing factors for IS. These results confirmed the negative single associations reported by Mórocz et al. [20].

In addition to predisposition to the development of IS, genetic factors could also influence the severity of the disease. The concept of disease-modifier genes as an element of genetic heterogeneity has been widely accepted and reported [21]. In the subgroup of surgical cases where Cobb angle was 40 degrees or higher the genotype and the allele frequencies of the* leptin* and* BMP4* promoter polymorphisms were comparable between cases and controls (*p* > 0.05). In conclusion, these polymorphisms alone could not be considered as modifying factors of IS associated with the progression of the deformity.

Adolescent idiopathic scoliosis is the most common spinal deformity [22] and the most frequently studied idiopathic scoliosis [2, 8–10, 20]. Our results revealed comparable genotype and allele distributions of the* leptin* and the* BMP4* promoter polymorphisms in the different age groups. These results suggest these polymorphisms alone could not be associated with the age of onset of the scoliosis.

Thoracic curve pattern is the most common in male and female patients as well as in surgical cases. In the subgroups of patients with different curve patterns no association between the promoter polymorphisms and the clinical phenotype was observed. In conclusion, the* leptin* and* BMP4* genetic variants were not associated with the curve pattern.

In clinical practice, about 90% of patients have been considered sporadic cases without familial recurrence. However the exact proportion of familial and sporadic idiopathic scoliosis is still unknown. In the subgroup of the familial cases, the* leptin* and* BMP4* genetic variants were not associated with the onset of the disease.

Scoliosis is more common in females than in males. In the subgroup of female patients no statistically significant association between the* leptin* and* BMP4* polymorphisms and the clinical phenotype was observed. In the small subgroup of male patients the frequency of the TT genotype of the* BMP4* polymorphic variant was two times higher than in the female sample and our results suggest a much larger case-control study will be needed to examine the role of this* BMP4* genetic variant in the etiology and pathogenesis of IS in males.

The results of the statistical analysis in this study indicate that the* leptin* -2548 G/A and the* BMP4* -3445 G/T functional polymorphisms were not significantly associated with susceptibility to IS or curve severity. On the basis of these results the examined polymorphic variants of* leptin* and* BMP4* genes could not be considered as genetic variants with predisposing effect or as a risk factor for the progression of the curve.

In addition, these results do not exclude synergistic effect of the promoter polymorphisms of* leptin* and* BMP4* in the etiology and pathogenesis of IS. Mórocz et al. [20] reported positive associations between* leptin*-*interleukin-6* and* BMP4-MMP3* genotype combinations and AIS. Their findings suggest that the effect of two or more predisposing genetic variants can be synergistic. In addition, there are probably other potential predisposing and modifying genetic variants of* BMP4* and leptin associated with the disease. The major limitation of our study is the small sample size which could affect the statistical power of the results.

## 5. Conclusions

In conclusion, this case-control study revealed no statistically significant association between the* leptin* (rs7799039) and the* BMP4* (rs4898820) functional polymorphisms and the susceptibility to IS or curve severity in Bulgarian patients.

No genotype or allele of the* leptin* and* BMP4* promoter polymorphisms was found to be correlated with age, gender, familial history, or curve pattern.

In the small subgroup of male patients the frequency of the TT genotype of the* BMP4* polymorphic variant was two times higher than in the female sample and our results suggest a much larger case-control study will be needed to examine the role of this* BMP4* genetic variant in the etiology and pathogenesis of IS in males.

The identification of molecular markers for IS could be useful for early detection and prognosis of the risk for a rapid progression of the curve. That would permit early stage treatment of the patient with the least invasive procedures.

## Figures and Tables

**Table 1:** Genotype and allele frequency distributions in patients (*n* = 90) with idiopathic scoliosis (IS) and healthy controls (*n* = 180).

Gene, polymorphism	Genotype, allele	Cases *n*, %	Controls *n*, %	*p* value
*Leptin* rs7799039	AA	25 (27.8)	55 (30.6)	0.64
	AG	42 (46.7)	88 (48.9)	
	GG	23 (25.6)	37 (20.6)	
	A	92 (51.1)	198 (55.0)	0.39
	G	88 (48.9)	162 (45.0)	

*BMP4* rs4898820	TT	27 (30.0)	46 (25.6)	0.74
	GT	46 (51.1)	99 (55.0)	
	GG	17 (18.9)	35 (19.4)	
	T	100 (55.6)	191 (53.1)	0.58
	G	80 (44.4)	169 (46.9)	

A *p*-value < 0.05 was considered to be statistically significant. *BMP4* indicates bone morphogenetic protein 4.

**Table 2:** Odds ratios of genotypes and alleles in different subgroups with idiopathic scoliosis (IS).

Subgroup	Gene, polymorphism	Genotype, allele	*p* value	OR [95% CI]
General (*n* = 90)	*Leptin* rs7799039	AA vs. GG A vs. G	0.38 0.39	1.37 [0.68–2.76] 1.17 [0.82–1.67]
	*BMP4* rs4898820	TT vs. GG T vs. G	0.62 0.58	1.21 [0.57–2.56] 1.11 [0.77–1.58]

AIS (*n* = 66)	*Leptin* rs7799039	AA vs. GG A vs. G	0.38 0.40	1.41 [0.65–3.03] 1.19 [0.80–1.77]
	*BMP4* rs4898820	TT vs. GG T vs. G	0.24 0.23	1.67 [0.70–3.98] 1.28 [0.85–1.91]

Familial history of IS (*n* = 25)	*Leptin* rs7799039	AA vs. GG A vs. G	0.23 0.23	2.08 [0.61–7.06] 1.43 [0.79–2.60]
	*BMP4* rs4898820	TT vs. GG T vs. G	0.19 0.15	3.42 [0.7–16.86] 1.57 [0.85–2.91]

Cobb angle >40° (*n* = 72)	*Leptin* rs7799039	AA vs. GG A vs. G	0.63 0.65	1.20 [0.56–2.58] 1.09 [0.74–1.61]
	*BMP4* rs4898820	TT vs. GG T vs. G	1 1	1.01 [0.46–2.26] 1.02 [0.69–1.50]

Thoracic scoliosis (*n* = 56)	*Leptin* rs7799039	AA vs. GG A vs. G	0.86 1	1.07 [0.47–2.45] 1.02 [0.66–1.56]
	*BMP4* rs4898820	TT vs. GG T vs. G	0.29 0.19	1.60 [0.67–3.82] 1.34 [0.86–2.07]

S-shaped scoliosis (*n* = 28)	*Leptin* rs7799039	AA vs. GG A vs. G	0.79 0.84	1.16 [0.40–3.38] 1.06 [0.60–1.86]
	*BMP4* rs4898820	TT vs. GG T vs. G	0.92 0.92	1.07 [0.31–3.64] 1.02 [0.58–1.80]

Males (*n* = 16)	*Leptin* rs7799039	AA vs. GG A vs. G	1 0.82	1.16 [0.21–6.56] 1.07 [0.45–2.50]
	*BMP4* rs4898820	TT vs. GG T vs. G	0.70 0.46	1.45 [0.28–7.64] 1.39 [0.58–3.37]

Females (*n* = 74)	*Leptin* rs7799039	AA vs. GG A vs. G	0.30 0.31	1.50 [0.69–3.25] 1.23 [0.83–1.82]
	*BMP4* rs4898820	TT vs. GG T vs. G	0.79 0.79	1.12 [0.48–2.63] 1.06 [0.71–1.57]

A value of *p* < 0.05 was considered to be statistically significant. *BMP4* indicates bone morphogenetic protein 4; AIS: adolescent idiopathic scoliosis; OR: odds ratio; CI: confidence interval.
